# ‘The air that we breathe’: assessment of laser and electrosurgical dissection devices on operating theater air quality

**DOI:** 10.1186/s40463-014-0039-1

**Published:** 2014-10-13

**Authors:** Matthew D Brace, Elizabeth Stevens, S Mark Taylor, Sarah Butt, Zhennan Sun, Licai Hu, Megan Borden, Neeraj Khanna, James Kuchta, Jonathan Trites, Robert Hart, Mark D Gibson

**Affiliations:** Department of Otolaryngology – Head and Neck Surgery; 3rd Floor Dickson Building, Victoria General Site; QEII Health Sciences Centre, 5820 University Ave, Halifax, Nova Scotia B3H 2Y9 Canada; Department of Process Engineering and Applied Science, Faculty of Engineering, Dalhousie University, Sexton Campus, 1360 Barrington Street, Halifax, Nova Scotia B3J 2X4 Canada; Faculty of Medicine – Dalhousie University, 5849 University Ave, CRC Building, Box 245, Halifax, Nova Scotia B3H 4R2 Canada

**Keywords:** Air quality, Surgical smoke, Plume, Ultrafine particles, PM_2.5_, Laser, Cautery

## Abstract

**Objectives:**

To measure changes in air quality during surgery.

**Methods:**

Operating room (OR) and hallway air quality was continuously monitored over a 3-month period. Rooftop monitoring was used to control for environmental changes and to account for the infiltration of outdoor air pollutants. Air quality measurements were correlated with operative times and electro-dissection equipment used.

**Results:**

OR air is cooler and drier compared to the adjacent hallway. Volatile organic compounds and other gases are below indoor air exposure limit guidelines. Lasers create greater 2.5 μm particulate matter (PM_2.5_) mass concentration, and greater fine and coarse particle number than cautery or cold tissue dissection. Cautery produces more ultrafine particles (UFP) than other dissection techniques. OR air has lower particle counts than outdoor environmental air by virtue of air conditioning HEPA filtration.

**Conclusion:**

Compared to the outside air, operating room air has lower particle counts. Lasers produce higher concentrations of PM_2.5_ mass and, fine and coarse particle number counts. Cautery produces higher concentrations of UFP number counts than other modalities and warrants consideration of the use of masks with ultrafine particle filtration capacity. Operating room air is consistently cooler with decreased humidity, which may cause airway irritation.

## Background

Modern surgical techniques employ a variety of electrosurgical dissection devices that provide means for efficient tissue dissection and maintenance of hemostasis. These devices include monopolar and bipolar electrocautery, ultrasonic scalpels, and a variety of lasers. A consequence of tissue dissection with these devices is the creation of surgical smoke. Surgical smoke results from rupture of cell membranes and vaporization of the intracellular contents [1]. In the case of electrocautery devices and lasers, this occurs by heating the tissues to their boiling point. The smoke produced by ultrasonic scalpels is created by compression of tissue on a rapidly oscillating plate that both cuts and coagulates tissue simultaneously creating a low-temperature vapor [2,3]. The smoke from all electrosurgical devices releases fine particulate matter into the operating room (OR) air [1,2]. Electrocautery devices produce the smallest particles with a mean aerodynamic size of 0.07 μm. Lasers produce larger particles with a mean diameter of 0.31 μm, and ultrasonic scalpels produce the largest particles ranging from 0.35-6.5 μm [3,4].

Increased concern has been raised regarding the health effects of surgical smoke on OR personnel [2,5-16]. Small particles carry chemical risks and larger particles have infectivity potential [3,4,10]. Surgical smoke inhalation is a known respiratory irritant and experience in an OR confirms its noxious odor [7,11]. There are a number of studies published examining the content of surgical smoke created by these devices [4,11,17-26]. Electrocautery dissection of tissue releases airborne hydrocarbons, nitriles, fatty acids, and phenols [3]. Lasers similarly release benzene, formaldehyde, acrolein, carbon monoxide, and hydrogen cyanide amongst other constituents [3,4,10,23,25-28]. The contents of ultrasonic scalpel vapor is not well studied or characterized [2,3,29].

Convincing studies have demonstrated the mutagenic potential of surgical smoke. These studies utilized standard Salmonella microsomal Ames tests [2,3,6]. However, the actual mutagenic risk to OR personnel is not known. Likewise, two studies have isolated viable tumor cells from surgical smoke. The actual risk to patients of tumor seeding, and risk to OR staff, again, is unknown [2,3]. HPV DNA has been isolated from both laser and electrocautery plume and successful culture of coagulase negative Staphylococcus, Corynebacterium and Neiserria from laser plume has been confirmed [2-4,6].

Interestingly, there is a small body of literature that has demonstrated that particle number counts in OR air can act as a surrogate for the quantity of airborne bacteria. These studies have shown a correlation between high counts of particles in the 5-7 μm range and surgical site infections [24,30]. However, correlation of particle number counting as a surrogate for airborne bacterial load is still controversial [20,31].

A particular concern with surgical smoke is the fine particle size and the potential respiratory effects [11]. A recent review of the literature reported particles in surgical smoke ranged in size from 10 nm to 25 μm. Particles smaller than 10 μm are inhalable, and UFP, less than 0.1 μm in diameter, become deposited in the alveoli where they are dependent on phagocytosis by alveolar macrophages for clearance [2]. The long-term health effects of UFP inhalation are unknown. Table 1 outlines Canadian indoor air quality exposure guidelines to these particles. Properly fitted standard surgical masks filter particles larger than 5 μm. Laser masks may filter particles as small as 0.1 μm. By definition, N95 masks filter 95% of non-oil based particles in the range of 0.1-0.3 μm [2,3].

**Table 1:** **Exposure limits**

	**Exposure Limits**
Fine particulate matter PM_2.5_	< 28 μg/m^3^ over a 24-hour period (2015 target) (CCME, 2012) [39]
UFP	1,000 – 20,000 particles/m^3^ (Air Quality Sciences, 2011)
CO	≤ 11.5 mg/m^3^ (≤11 ppm) over a 24 hour period (Health Canada, 2012) [40]
	≤ 28.6 mg/m^3^ (≤25 ppm)over a 1 hour period (Health Canada, 2012) [40]
NO_2_	≤ 100 μg/m^3^ (≤0.05 ppm) over a 24 hour period (Health Canada, 2012) [40]
	≤ 480 μg/m^3^ (≤0.25 ppm) over a 1 hour period (Health Canada, 2012) [40]
SO_2_	≤ 50 μg/m^3^ (≤0.019 ppm) (Health Canada, 2012) [40]
Fine particle number count*	-
Coarse particle number count*	-
Relative humidity	30% to 55% (Health Canada, 2008) [37]
Indoor temperature	17 to 27°C (Balaras et al., 2006) [38]
CO_2_	≤ 6300 mg/m^3^ (≤3500 ppm) (Health Canada, 2012) [40]

Canadian indoor air quality exposure limit guidelines. *No current guidelines exist for ultrafine, fine, or coarse particle number counts.

Clearly there is cause for concern for all hospital staff employed in the OR and patients alike. This has resulted in both British and American national workplace recommendations for the use of local exhaust ventilation systems for the evacuation of surgical plume during surgery in addition to existing OR ventilation systems [15,18,32]. Additionally, the Canadian Centre for Occupational Health and Safety has published guidelines for laser plume safety [27]. Manufacturers of electrosurgical dissection devices have responded by creating devices with built-in smoke evacuators, however, studies indicate that these devices are often cumbersome for surgeons, and are therefore often not used [2,8-10].

While studies employing direct sampling and analysis of surgical plume have been performed, there is little literature that examines the actual daily changes in OR air quality [16]. Most studies surround interval particulate counting and surgical site infections in orthopedic surgery [24,30,31]. Current World Health Organization guidelines do exist for indoor workplace air quality standards [33] and the American Society of Heating, Refrigerating, and Air Conditioning Engineers have published similar guidelines [34]. The goal of this study was to monitor the daily changes in air quality in the Otolaryngology-Head and Neck Surgery OR to determine if, and by what degree, OR air quality changed during surgeries employing different tissue dissection devices. Specifically, this study examined changes in air quality with respect to the use of lasers compared to other electrosurgical dissection devices during surgery.

## Methods

Ethics approval for this study was deemed unnecessary by Capital Health Halifax’s ethics department as only air quality measurements were made and no patient data was collected. The study took place at the Victoria General Hospital in Halifax, Nova Scotia, Canada. The Otolaryngology-Head and Neck surgery service operates primarily out of 2 dedicated rooms opposite of each other. The full spectrum of Otolaryngology operative cases is carried out in these rooms. These surgeries routinely require the use of electrocautery, carbon dioxide (CO_2_) and potassium titanyl phosphate (KTP) lasers, as well as ultrasonic scalpels.

### Monitoring

Continuous monitoring was conducted from November 5 to November 30, 2012, and from February 6, 2013 to April 2, 2013. There were three monitoring locations. Monitoring equipment was placed in the OR. A second identical monitoring setup was placed in the hallway adjacent to the OR. Outdoor air quality was monitored on the roof of an adjacent Dalhousie University building (east of the hospital at a distance of 900 m) to control for outdoor meteorological changes and to account for the infiltration of outdoor air pollution into the OR and adjacent hallway. Equipment was positioned on shelves and on sealed windowsills to facilitate continuous room monitoring without obstructing the daily use of the OR. Parameters measured included temperature, relative humidity, CO_2_, carbon monoxide (CO), hydrogen sulfide (H_2_S), ammonia (NH_3_), oxygen (O_2_), median aerodynamic diameter particles less than, or equal to, 2.5 μm (PM_2.5_), UFP number counts (size range: 0.02 – 0.1 μm), fine (0.1 - 2.5 μm) and coarse (2.5 – 10 μm) particle number counts. These measurements were carried out utilizing air quality equipment including TSI DustTrak™ 8520 PM_2.5_ monitor (TSI Incorporated, Shoreview, MN 55126, US) TSI P-Trak™Ultrafine Particle Counter (TSI Incorporated, Shoreview, MN 55126, US), ppbRAE Plus™ Monitor (RAE Systems), Critical Environment Technologies YES 206 Falcon™ monitor (Critical Environment Technologies Canada Inc, Delta, BC, V4G 1 M3, Canada), and Dylos™ Corporation DC1700 Battery Operated Air Quality Monitor (Dylos Corporation, Riverside, CA 92504, US). Details of the air quality equipment used are outlined in Table 2. The air quality equipment was maintained daily by a group of students from Dalhousie University, Faculty of Engineering.

**Table 2:** **Air quality measuring equipment**

**Device**	**Metrics measured**
DustTrak™ Aerosol Monitor 8520	PM_2.5_
P-Trak™ Ultrafine Particle Counter	UFP
ppbRAE Plus™ Monitor	volatile organic compound (VOC)
VRAE Monitor™	CO, H_2_S, NH_3_ and O_2_
YES 206 Falcon™ Monitor	CO_2_, temperature and relative humidity
Dylos DC1700™ Air Quality Monitor	Fine particle number count, Coarse particle number count

### Surgical case details

Daily elective surgical lists were collected for the study period. Waitlist and after hours cases were identified from the OR case logs. Intraoperative records were used to determine electrosurgical dissection equipment used as well as operative start and stop times. These times were cross-referenced to air quality measurements for analysis.

### Data analysis

Data from each device at each monitoring site were compared for readings taken during surgery. Cases were divided to compare procedures utilizing lasers vs procedures utilizing all other electrosurgical devices vs procedures utilizing cold tissue dissection. Analysis was performed using non-parametric Kruskal-Wallis one-way analysis of variance employing a Dunn’s test for multiple comparisons. A Kolmogorov-Smirnov test of normality was performed on the data. SigmaPlot statistical software (Systat Software, San Jose, CA) was employed for all calculations.

## Results

The analysis of results will focus on PM_2.5_ mass concentrations, UFP, fine and coarse particle counts, temperature, relative humidity, and CO_2_. Though NH3, H2S, CO, and VOC were measured; they are outside the scope of this paper.

### Study cases

Overall, air quality measurements were conducted for 146 surgical cases over 57 days during a study period of 80 days. A total of 40 cases did not use laser or cautery devices. In total, air quality measures from 90 cases utilizing electro-dissection devices were examined. Of these, 25 cases utilized lasers; 18 were CO_2_, 4 were KTP, and 3 were 980 diode lasers. The remaining 65 cases utilized bipolar and monopolar cautery, with 6 cases also employing the harmonic scalpel. A total of 16 cases had no record of device used.

### Temperature, relative humidity, and gases

**Figure 1:**
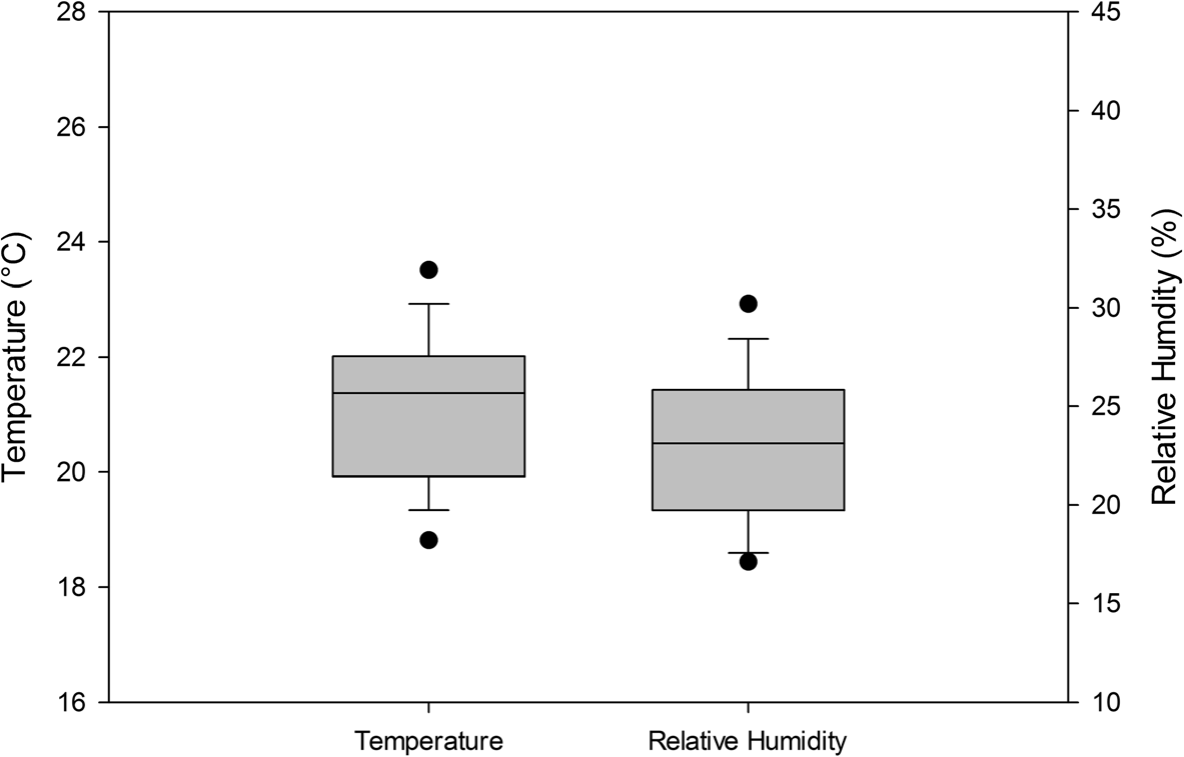
**Mean OR temperature and relative humidity during all procedures.**

**Figure 2:**
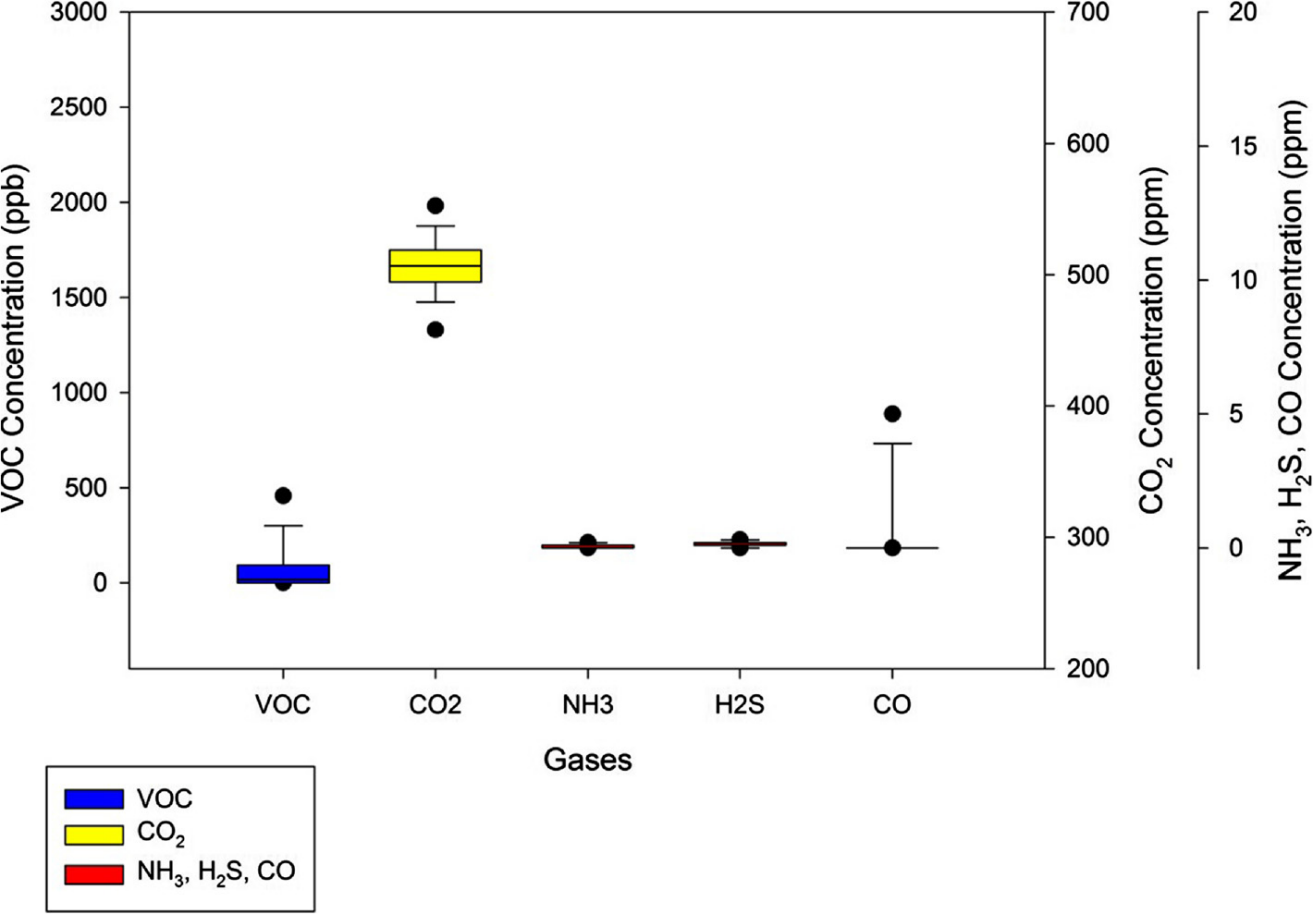
**Mean OR gas measurements during all procedures.** O2 = oxygen, VOC = volatile organic compounds, CO2 = carbon dioxide, NH3 = ammonia, H2S = hydrogen sulfide, CO = carbon monoxide.

Mean values for OR air temperature and humidity are depicted in Figure 1. CO_2,_ CO, NH_3_, and H_2_S levels during surgery are shown in Figure 2. The average indoor temperature (OR and hallway) was 20.4 to 23.0°C. The percent relative humidity averaged 26% in the hallway, but in the OR ranged from 21.9 to 23.8%. OR carbon dioxide levels averaged 446.2 ppm, mean CO levels were 0.81 ppm, mean NH_3_ measured 0.87 ppm, mean H_2_S measured 0.17, and mean O_2_ measured 20.9%.

### Particle counts

**Figure 3:**
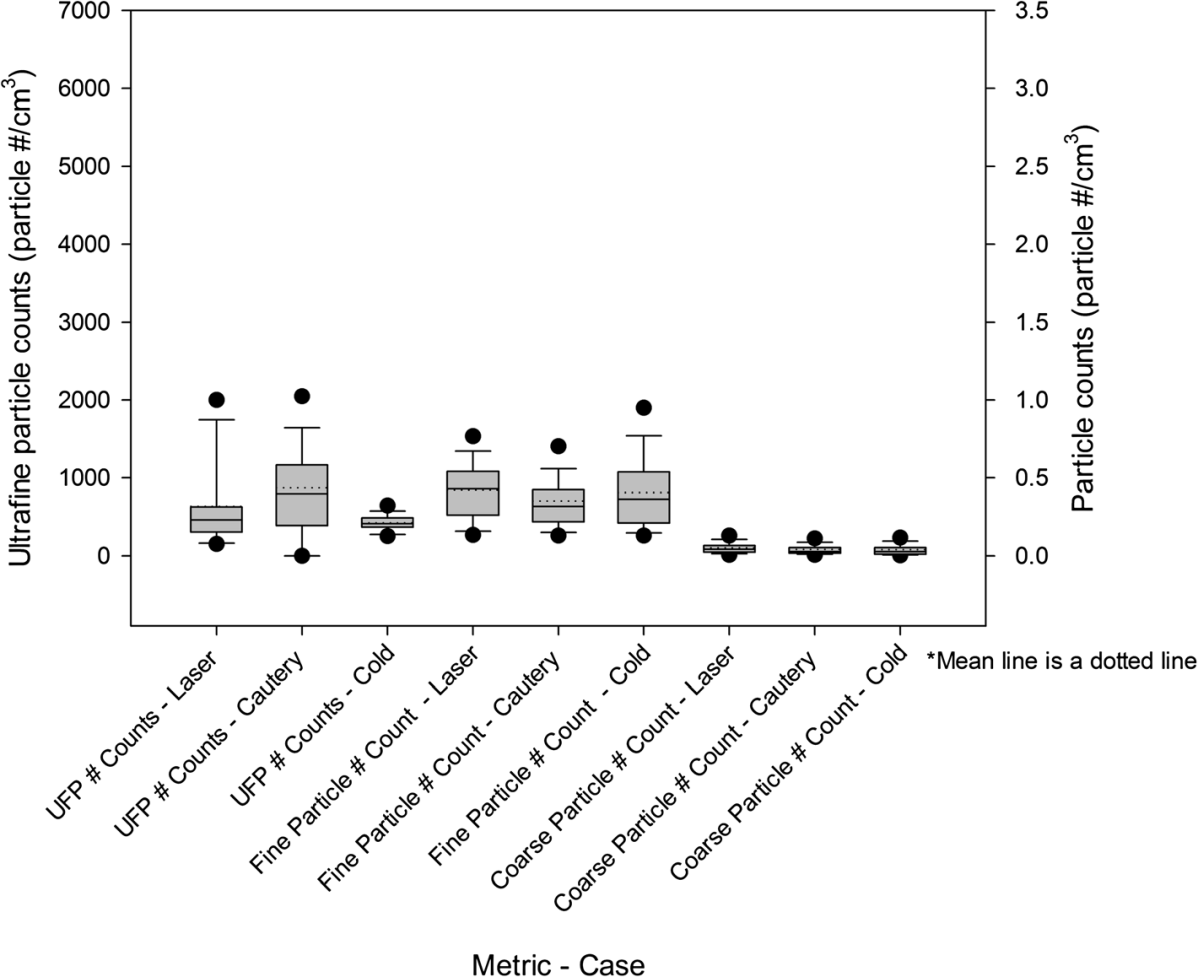
**Median and mean OR particle counts during surgery.**

**Figure 4:**
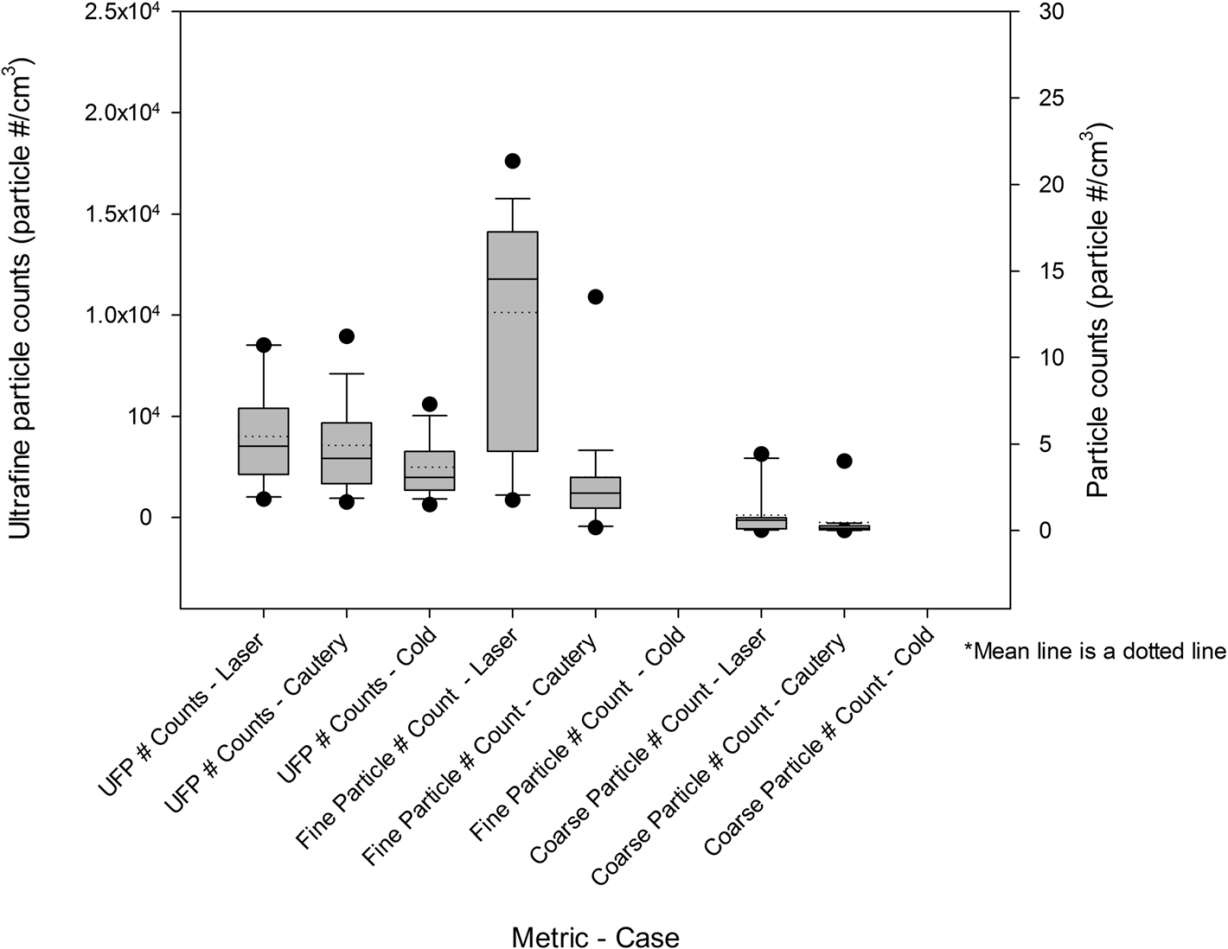
**Median and mean rooftop outdoor particle counts during surgery.**

Data are displayed in Tables 3, 4, and 5. Normality tests for all data sets revealed non-parametric distributions. A Kruskal-Wallace test with a Dunn’s sub-test for multiple comparisons was performed (α = 0.05). Figures 3 and 4 depict OR and environmental particle counts respectively.

**Table 3:** **Analysis of variance results comparing laser and cautery particle counts**

**Metric**	**Location**	**Laser median**	**Cautery median**	**ANOVA results**
PM_2.5_ (μg/m^3^)	Hallway	2.00	2.00	P < 0.05
	OR	3.00	2.00	P < 0.05
	Roof	23.0	22.0	P > 0.05
UFPs (particle #/cm^3^)	Hallway	564	668	P > 0.05
	OR	462	798	P < 0.05
	Roof	14105	11646	P > 0.05
Fine particle number count (particle #/cm^3^)	Hallway	0.45	0.42	P > 0.05
	OR	0.43	0.32	P < 0.05
	Roof	14.53	2.17	P < 0.05
Coarse particle number count (particle #/cm^3^)	Hallway	0.07	0.06	P < 0.05
	OR	0.04	0.03	P < 0.05
	Roof	0.60	0.13	P < 0.05

PM = particulate matter, UFP = ultrafine particle.

**Table 4:** **Analysis of variance results comparing laser and cold dissection particle counts**

**Metric**	**Location**	**Laser median**	**Cold dissection median**	**ANOVA results**
PM_2.5_ (μg/m^3^)	Hallway	2.00	2.00	P >0.05
	OR	3.00	2.00	P < 0.05
	Roof	23.0	9.00	P > 0.05
UFPs (particle #/cm^3^)	Hallway	564	655	P > 0.05
	OR	462	415	P > 0.05
	Roof	14105	7902	P < 0.05
Fine particle number count (particle #/cm^3^)	Hallway	0.45	0.42	P > 0.05
	OR	0.43	0.36	P < 0.05
	Roof	14.53		
Coarse particle number count (particle #/cm^3^)	Hallway	0.07	0.06	P < 0.05
	OR	0.04	0.03	P < 0.05
	Roof	0.60		

Roof top outdoor counts were unavailable for coarse and fine particles during cold dissection. PM = particulate matter, UFP = ultrafine particles.

**Table 5:** **Analysis of variance results comparing cautery and cold dissection particle counts**

**Metric**	**Location**	**Cautery median**	**Cold dissection median**	**ANOVA results**
PM_2.5_ (μg/m^3^)	Hallway	2.00	2.00	P < 0.05
	OR	2.00	2.00	P < 0.05
	Roof	22.0	9.00	P > 0.05
UFPs (particles/cm^3^)	Hallway	668	655	P > 0.05
	OR	798	415	P < 0.05
	Roof	11646	7902	P < 0.05
Fine particle number count (particle #/cm^3^)	Hallway	0.42	0.42	P > 0.05
	OR	0.32	0.36	P < 0.05
	Roof	2.17		
Coarse particle number count (particle #/cm^3^)	Hallway	0.06	0.06	P < 0.05
	OR	0.03	0.03	P > 0.05
	Roof	0.13		

Roof top outdoor counts were unavailable for coarse and fine particles during cold dissection. PM = particulate matter, UFP = ultrafine particle.

### Laser cases vs Cautery cases

A significantly elevated PM_2.5_ mass concentration (P < 0.001), fine particle (P < 0.001), and coarse particle number counts (P < 0.001) were observed in the OR during laser cases compared to cautery cases. This difference was also observed in the adjacent hallway for PM_2.5_ (P < 0.001) and coarse particle number (P < 0.001). There were no corresponding significant changes in outside environmental PM_2.5_ or UFP number counts. Outdoor coarse (P < 0.001) and fine particle number counts (P < 0.001) were significantly greater during laser cases compared to cautery cases. The UFP number counts were significantly higher during cautery (P < 0.001) cases compared to the laser cases.

### Laser cases vs cold dissection cases

A significantly higher count of PM_2.5_ (P < 0.001), fine (P < 0.001) and coarse (P < 0.001) particle number was observed in the OR during laser cases compared to cases utilizing no cautery or laser. There was no difference in UFP counts in the OR. UFP were significantly elevated outdoors during laser cases compared to no laser or cautery cases (P < 0.001). Coarse particle number counts were significantly elevated in the hallway during laser cases compared to cases with no cautery or laser (P < 0.001).

### Cautery cases vs cold dissection cases

Significantly higher PM_2.5_ mass concentration (P < 0.001), UFP (P < 0.001), and fine particle number counts (P < 0.001) were observed in cautery cases compared to those cases utilizing no cautery or laser. A significantly higher PM_2.5_ mass concentration was also observed in the hallway during cautery cases (P < 0.001). A significantly higher count of coarse particles was observed in the hallway during cautery cases.

## Discussion

Air quality has become an important concern for health care workers employed in the OR. Surgical smoke is created through the routine use of energy driven tissue dissection including cautery devices, ultrasonic scalpels, and lasers. The size of the particles created varies between devices [3,4]. A number of reports have described the contents of surgical smoke as airway irritants, mutagens, and a potential vehicle for the spread of malignancies or infection [2-16]. While OR air filtering and exchange systems do exist, many feel these are inadequate and additional smoke evacuation devices have been introduced. Unfortunately, these devices are often cumbersome to use or bulky to handle and often are not employed by surgeons.

Surgical masks afford an additional level of protection from aerosolized contents of surgical smoke. However, the particulate filtering efficiency differs between masks with respect to particulate size. The N95 masks provide the greatest level of mask filtration, and it requires individual fitting for optimal performance. These masks give 95% filtration of particles in the 0.1-0.3 μm, however, it is incapable of filtering all UFPs. Standard surgical masks will not filter UFP, PM_2.5_, or fine particles, only particulate matter greater than a median aerodynamic diameter of 5 μm. Laser masks can filter particles as small as 0.1 μm, with up to 99% particulate filtration efficiency when worn properly and changed regularly. However, they do not filter UFPs [2].

The use of laser techniques in Otolaryngology-Head and Neck Surgery has increased exponentially in the past 4 decades [35]. Trans-oral laser microsurgery of the larynx and oropharynx accounts for a growing proportion of laser cases at our institution. Although this technique has many advantages, one disadvantage is the surgical plume created and the difficulty achieving reliable smoke evacuation. In many cases, we find that the smoke evacuation system is inadequate and that surgical plume obscures the view through the laryngoscope. This necessitates a surgical pause to manually suction the field. Our concern has been that during the dissection, much of the smoke escapes into the OR where it may be inhaled by the OR personnel. Given the health concerns related to surgical smoke, we endeavored to obtain measures of OR air particles as a measure of air quality during cases that involved laser dissection to compare to cautery and cold dissection cases.

This study demonstrated that the use of lasers in the OR was associated with an increased concentration of measurable PM_2.5_, fine, and coarse particle number counts compared to the use of cautery or cold dissection techniques. While these results did reach statistical significance, there was a large amount of overlap in the counts between surgical modalities. This is likely a by-product of the study design. No two surgeries utilized the same degree or duration of tissue dissection. In addition, cases designated as laser cases were variably associated with neck dissections utilizing cautery and or ultrasonic equipment. It was not possible to separate these cases into component parts and as such, the data is not completely clean. Further investigations to sample the smoke from individual devices are currently underway.

There was a significant increase in the coarse and fine particle number counts during the laser cases compared to cautery and cold dissection cases, but this was associated with a significant shift in the outdoor counts of the same particle size fractions. A firm conclusion regarding the laser production of coarse particle number counts compared to other techniques is not possible without further investigation.

The use of cautery appears to be associated with the liberation of significantly increased levels of UFPs compared to laser and cold dissection cases. This is the most important finding of this study. UFP have been linked to respiratory disease with evidence demonstrating increased exacerbations of asthma corresponding to increased environmental UFP counts [36]. The long-term effect of exposure to these particles is unknown. Currently in Canada there are no exposure guidelines for UFPs except to minimize exposure. For surgical cases utilizing cautery devices, the standard surgical masks will not filter any of the UFPs. At minimum, it would be prudent for OR personnel to employ laser masks during procedures utilizing cautery to filter a portion of the UFPs.

The level of UFPs measured in the OR was significantly lower than that measured in the outside air. This observation was true for all measured particles. The OR environment sampled in this study is equipped with a filtered air exchange system that provides 18-20 air exchanges per hour. This obviously plays an important role in not only filtering the products of surgery out of the OR, but in filtering the air supplied to the OR as well. Overall, the air quality in terms of particle counts was better in the OR than the outside air. In addition, all measured gases were well below recommended exposure limits [37-39]. However, relative humidity was low in both the OR and hallway. Compared to the recommendation of 30-55% relative humidity by the Health Canada 2008 Indoor Air Quality Guidelines [37], the OR air ranged 22-23%, while hallway air had a relative humidity of 26%. While these measures are low by indoor air standards [38], they do fall within the accepted range of 20-60% for health care facilities recommended by the American Society of Heating, Refrigerating, and Air Conditioning Engineers Standard [34,40]. For OR personnel, this may lead to airway irritation from dryness, independent of chemical or particulate content of the air.

## Conclusion

Air quality measurements in the OR show smaller particle number counts than outdoor air. The temperature is cooler in the OR than in adjacent hallways. The relative humidity in the OR is much lower than standard indoor air humidity. This may cause airway drying and irritation. Laser tissue dissection appears to increase the PM_2.5_ mass concentration and fine particle number count. In addition, lasers appear to also increase coarse particle number counts. Electrocautery tissue dissection increases the counts of UFPs more than laser dissection. Until data on personal exposure monitoring is available, the use of surgical masks with UFP filtration capacity warrants consideration by surgeons not only during cases employing lasers but those employing standard cautery as well. Further investigation to specifically sample smoke products from individual instruments is underway.

## Abbreviations

CO: Carbon monoxide
CO_2_: Carbon dioxide
H_2_S: Hydrogen sulfide
NH_3_: Ammonia
O_2_: Oxygen
OR: Operating room
PM_2.5_: Particulate matter 2.5 μm
UFP: Ultrafine particle
